# Attempted Femicide: A Mixed Methods Approach To Deriving An Operational Definition for the Fedisa Modikologo Study, South Africa

**DOI:** 10.1007/s10896-026-01075-4

**Published:** 2026-02-26

**Authors:** Rachel Jewkes, Shibe Mhlongo, Esnat Chirwa, Leane Ramsoomar, Samantha Willan, Nataly Woollett, Nicola Christofides, Boitumelo Seepamore, Darshini Govindasamy, Jani Nöthling, Venice Mbowane, Maureen Phakoe, Desire Pass, Amanda Zembe, Charntel Paile, Laura Washington, Nwabisa Shai, Mercilene Machisa, Pinky Mahlangu, Stanley Carries, Naeemah Abrahams

**Affiliations:** 1 Gender and Health Research Unit, South African Medical Research Council, Pretoria, South Africa; 2 Office of the Executive Scientist, South African Medical Research Council, Pretoria, South Africa; 3 School of Public Health, Faculty of Health Sciences, University of the Witwatersrand, Johannesburg, South Africa; 4 Biostatistics Unit, South African Medical Research Council, Pretoria, South Africa; 5 School of Health Systems and Public Health, University of Pretoria, Pretoria, South Africa; 6 The School of Applied Human Sciences (Social Work), University of KwaZulu-Natal, Durban, South Africa; 7 Health Systems Research Unit, South African Medical Research Council, Durban, South Africa; 8 Diakonia Centre, Project Empower, Durban, South Africa; 9 School of Nursing and Public Health, University of KwaZulu Natal, Durban, South Africa; 10 School of Public Health and Family Medicine, University of Cape Town, Cape Town, South Africa

**Keywords:** Femicide, Attempted femicide, Methods, Definition, South africa, Murder

## Abstract

**Purpose:**

Women often do not recognize when they experience attempted femicide, so another way of measuring it is needed for research. We examined narrative accounts of femicide attempts, and analysed data to determine whether we could systematically expand the definition of attempted femicide, for the purposes of research.

**Methods:**

We recruited a non-probability sample of 3742 women into the Fedisa ModikologoStudy. They were 18–45 years and had experienced severe intimate partner violence in the previous year. They completed an interview with two closed and one open question about attempted femicide experiences.

**Results:**

Strangulation, suffocation, stabbing, bludgeoning, gun shots, fire, mocked suicides and accidents were all used in attempted femicides, which 32.4% (1211/3742) of women said they had experienced. These were significantly more common among this sub-group of women. Gunshot injuries and loss of consciousness due to strangulation were the most strongly associated with a recognized femicide attempt, however other injuries were also associated. Assuming unrecognized femicide attempts were broadly similar, we conducted an analysis with recognized femicide attempts as a putative gold standard, and examined change in the sensitivity, specificity and ROC AUC of including different elements in the attempted femicide definition. After incrementally adding elements, we optimized the model with a sensitivity to 85.0%, specificity of 52.7% and ROC AUC of 67.1.

**Conclusions:**

Our findings point to a working definition of attempted femicide as having a recognized femicide attempt, or injuries leading to loss of consciousness, acts of strangulation, suffocation, gunshots, burns, or stabbing to the neck, torso or genitals.

## Introduction

In South Africa, interpersonal violence is the second most highly ranked risk factor for the country’s years of life lost in women (Bradshaw et al., 2022). The country’s National Femicide Study, based on research from mortuary and police data, reported an intimate partner femicide (IPF) rate of 4·6/ 100 000 population in 2017 (Abrahams et al., 2024). This rate appears particularly high when compared to those reported in many other countries The United Nations Office on Drugs and Crime (UNODC), reported that in 2023 there was a global rate of femicide by intimate partners and family members of 1·3/100 000 population, amounting to 60% of female murders globally (UNODC and UN Women, 2024). In the United States of America (USA), femicide has been the leading cause of death of African American women aged 15–34 years (Grisso et al., 1999). However, most of the global statistics are not reliable as most are based on administrative data, which substantially underestimate the prevalence of IPF. In most countries health, police, social services and criminal justice data systems are not linked and police data have substantial gaps in reported perpetrators of women killed (Zecha et al., 2023).

In South Africa, the most recent annual national police statistics indicate that in 2023/4 there were 5578 reported murders of women, and 7239 attempted murder cases and 63 054 cases of assault with grievous bodily harm opened by the police (Crime Registrar Head Office, 2024). In South African law, assault with grievous bodily harm and attempted murder are not defined in a way that is specific enough to be used in research as the difference between the two depends only on whether intent to kill can be proven, and intention is a highly contested area. ‘Intent’ has not been included in femicide reporting (United Nations Office on Drugs and Crime, 2023), as the focus is rather on whether the killing occurred, irrespective of intent. Attempted intimate partner femicide generally is not defined. We regard it as the performance of acts or the deliberate causing of injury to a woman by a current or former intimate partner, or would-be partner, or that were intended to, or were of such a severity that they might have, resulted in the death of a woman, but did not do so. There is a need to operationalize such a definition of attempted IPF so that can be used in research, specifying life-threatening acts performed, and injuries, if there is to be a systematic study of the phenomenon.

Campbell and colleagues used a definition of attempted femicide when they conducted research in the USA to identify risk factors for IPF and attempted femicide (J. C. Campbell et al. 2003a, 2003b). They defined attempted femicide as having survived “a gunshot or stab wound to the head, neck, or torso; loss of consciousness from strangulation; trauma, or attempted drowning; other severe injuries that could have led to death; and/or verifiable evidence of unambiguous intent to kill the victim”(J. C. Campbell et al. 2003a, 2003b). The research was under-taken in conjunction with law enforcement officials, so the ‘verification’ of evidence was conducted during police investigations (J. C. Campbell et al. 2003a, 2003b). Lewandowski et al. conducted research with children who had witnessed attempted femicide or femicide and they used a similar definition : “a gunshot or puncture (stab) wound to the head, neck, or torso; loss of consciousness from strangulation; multiple blows to the head with a blunt object (excluding cases where the object could not normally have resulted in death); and unambiguous evidence of an attempt to kill (recorded or with a credible witness) (Lewandowski, 2004).” A further study was qualitative, and conducted in the United States as a study linked to that of Campbell et al., and so it used much the same definition of attempted femicide (Nicolaidis et al., 2003). A key finding was that half of the women interviewed had not recognized that they were in danger of being killed. Since this early work in the United States, interest in femicide and attempted femicide has grown internationally and three recent systematic reviews published have included between 17–28 studies (Garcia-Vergara et al., 2022; Matias et al., 2020; Spencer & Stith, 2020). These highlight both limited research on attempted femicide and the fact that a unified definition has not been developed. Many researchers rely on identification of such cases through police, courts or health services records, or self-identification by women (Avieli, 2024). Following Nicolaidis et al.’s observation that many women do not recognize the severity of violence they experience (Nicolaidis et al., 2003), an injury-based definition of attempted femicide, combined with recognized accounts of potentially lethal acts performed, is likely to provide the most reliable approach to defining attempted femicide.

### Injuries in IPF Cases

The South African national femicide studies collect victim injury data from post-mortem reports, and an in-depth analysis of the profile of injuries was conducted as part of the 1999 study (Abrahams et al., 2011; Mathews et al., 2009). The main mechanisms of death of women from IPF are gun shot, sharp injuries and blunt force injuries, with strangulation, asphyxiation and burns also reported, although less frequently (Abrahams et al., 2009). In addition, about 16% of murdered women had been raped, commonly before strangulation. Analysis of the location of fatal injuries shows that they were overwhelmingly to the head and neck, or torso, with some variation per mechanism of death, for example, neck injuries were predominant with strangulation (in 95% of cases and head injuries found in 95% of deaths by blunt force (Mathews et al., 2009). In 82% of deaths by a sharp object, there was injury to the thorax (Mathews et al., 2009).

The Fedisa Modikologo Study, in South Africa, aims to study IPF to understand more about the context in which it occurs, to unravel the critical question of which women are more at risk of IPF, and thus advance our understanding of how it may be prevented. The research will track women experiencing severe IPV recruited through four sites across the country and follow them up through the National Population Database, which registers deaths. The working definition of intimate partner femicide for the study will be the murder of a woman by an intimate partner (i.e. a current or ex-husband/boyfriend, same-sex partner or a rejected would-be partner). The term ‘murder’ is used in the country to refer to the intentional killing of a person. However, even in the country with the highest femicide rate in the world, attempted femicides are much more frequent than actual femicides, and so a second outcome for the study is proposed to be ‘attempted intimate partner femicide’. Thus, an important question is how these should be identified and defined.

The aim of this paper is to describe the process by which we developed an operational definition of attempted femicide for use in research as an outcome variable. The objectives of the paper are to present accounts of attempted intimate femicide provided by women interviewed, and to describe how we determined how attempted femicide should be defined for the study through a statistical analysis of reports of attempted femicide and information on injuries, potentially lethal acts and hospitalization.

## Methods

The sample was recruited as a non-probability sample in four Fedisa Modikologo research sites in South Africa: the Western Cape, KwaZulu-Natal, Gauteng and Limpopo Provinces. Our inclusion criteria were intended to enroll women at particularly high risk of intimate femicide. Participants recruited into the study were women aged 18–45 years, who identified as female at birth or subsequently self-identified, and who had a current or former male partner who subjected them to severe intimate partner violence (IPV). In the case of an ex-partner, the relationship must have ended in the 12 months preceding the interview, and the partner should still be feared, intimidating, stalking or contacting her in a way she found threatening. The operational definition of severe IPV was that she would been hit with a fist, or something that could hurt, strangled, kicked, dragged, beaten up or threatened with/injured by a weapon; or been raped or forced into sex, or engage in sexual acts against her wishes; or have had threats to kill her, threats to harm any children in order to hurt her, or stalking, and she should fear him. Alternatively, she may have experienced multiple forms of severe controlling behavior, such as being confined to the home, prevented from working or earning an income, or experienced jealous surveillance. Further details of the methods for the Fedisa Modikologostudy are described in a methods paper (Jewkes et al., 2025).

Participants were referred to the study by formal and informal services, such as non-governmental organisations and individuals in the vicinity of the study sites who knew of women experiencing severe IPV, as well as by participant chain referral (a variation on respondent-driven sampling). On completion of an interview, participants were asked if they knew other women in the same situation who may be willing to participate in the research. If so, they were given a study information leaflet and up to three coupons with the contact details of the research site for the other women, and a request that they be asked to make telephonic contact with the research site. The analysis used baseline data from the Fedisa Modikologocohort which was still actively recruiting. We based the analysis on interviews with the first 3742 participants enrolled in the study across the four sites.

Potential participants were screened for participation at our study sites and those eligible were enrolled after completing written informed consent procedures. Those who consented, completed an interview that lasted 1.5–2 h, with data recorded on REDCap (a web-based survey platform). They were given R200 (~ £8.50) plus travel expenses for the interview. All participants were offered counselling by one of the fulltime study social workers, provided with basic containment support by interviewers, as needed, and offered any other referrals that were indicated or requested, including, at times, access to shelters, or help opening a case with the police.

## Measures

### Direct Measure

The questionnaire had three questions that asked women directly about whether they had ever been subjected to an attempted murder. These were: (1) Do you think there was an occasion when __(name)_________ actually tried to kill you and did not succeed? Response categories were yes/no. (2) Has anything ever happened that made you think __(name)_________ may have been trying to kill you but you were not certain that he was the cause, for example a fire at home that you think he may have caused deliberately, or strangulation or suffocation, or your brakes failing on the car, or an assailant coming into the home to assault you that he may have arranged, or anything else? Four-point response categories were used with ‘this definitely happened’, and degrees of lesser certainty to ‘this did not happen’. We considered ‘this definitely happened’ as being indicative of attempted murder. (3) An affirmative response to the questions led to a follow-up open-ended item asking for details of what happened. For this analysis, answering ‘yes’ to the first question, or ‘this definitely happened’ to the second, was considered to indicate that a woman had experienced attempted femicide. Some of the quotes were recorded verbatim and others paraphrased by the interviewer.

### Indirect Measure

#### Acts, Injuries and Hospitalization Indicative of Attempted Femicides

Recognizing that women may have been subjected to an attempted murder without being fully aware that they could have lost their lives, we also examined responses to questions about ever having experienced acts and injuries that may indirectly indicate attempted femicide.

After a screening question on whether the participant had been injured, and how often, by her primary partner, the questionnaire asked about types of injuries (burns, smothering, strangulation, gunshot, stabs or cuts, bruises, broken bones or teeth) and the bodily location of injuries. There was a question of ever having lost consciousness due to head injury with response categories (brief, 1–30 min, 31 min to 24 h and over 24 h). A question was asked about having ‘ever lost consciousness due to strangulation’. Finally, having stayed overnight in a hospital or clinic due to injuries by the partner in the past year, and the number of nights. We used these items in the analysis to potentially indicate attempted femicide.

### Data Analysis

We analysed the qualitative data by extracting the responses to the open-ended questions about what the participant’s partner had done when she had experienced a femicide attempt into an Excel spreadsheet. We then grouped descriptions thematically and constructed an account that gave an overview of the themes, drawing out illustrative quotes.

For quantitative analysis, we used descriptive statistics (frequencies with percentages and means with standard deviations) to describe the overall sample. We summarized the responses to the questions about whether the participant had experienced an attempt on her life by her partner, whether she thought a life-threatening situation she experienced was definitely caused by him, and the combination of the two that make up what we refer to as the direct femicide attempt.

We calculated the percentages (column or row) of women who experienced any potentially life-threatening injuries or acts due to their partner’s violence, and whether they had been hospitalized for these, by whether or not they recognized themselves as having had an attempted femicide experience. We report on the crude odds ratio (OR) with 95% confidence intervals (CIs) of the attempted femicide against the indirect methods. The series of acts and injuries considered were guided by the narratives of attempted femicide, as well as injuries described at autopsy in femicides, and medical knowledge of acts that may be life-threatening, and in the case of hospitalization, indicative of greater severity (Mathews et al., 2009). We derived a variable for experiences of stabbing or cuts to the neck, genitals or torso. These body parts were chosen due to the close connection between genital injury and femicide, as well as both the lethal potential of neck and torso stabs (Abrahams et al., 2008; Mathews et al., 2009). Further, the fact that ‘cuts’ are only found in the neck and torso as a result of very severe violence, and/or use of a sharp object.

Thereafter we calculated the proportion of women in the sample who would have been viewed as having an ‘attempted femicide experience’ for each definition, as the number of elements was incrementally increased. Next, we fitted a multivariate logistic regression model by including all acts and injuries against attempted femicide experience to assess key injuries or acts. We report the adjusted odds ratio (aOR) and their 95% CIs.

Finally, we assumed that a woman’s recognition that there had been an attempt on her life could be viewed as a ‘gold standard’ for attempted femicide. To examine the predictive power of the indirect plus direct definition, we compared a series of indirect definitions against the gold standard. We calculated the sensitivity (a test’s ability to correctly identify individuals with the condition - i.e. true positives), specificity (a test’s ability to correctly identify individuals without a condition – i.e. true negatives), positive predictive value (PPV) and negative predictive value (NPV) and the receiving operating characteristics (ROC) area under curve (AUC) of each different definition of attempted femicide. Each successive model had a definition with one additional element included. The best definition was the one that would give us the highest level of sensitivity and specificity and highest ROC AUC, recognizing that there if often a trade-off between optimized sensitivity and specificity.

As a sensitivity analysis we considered whether the sensitivity and specificity would be improved by not including burns, recognizing them not to be always life-threatening. We also tested whether having hospitalization restricted to two or more nights made a difference, and whether having bruises with hospitalization, was better than just having bruises. We also tested some variations in the order variables were entered into the models. We found none of these changes to improve the ROC over models 7 and 8 and do not present the findings. We conducted reliability tests to determine whether the timepoint of examining the data influenced the findings of the analysis, since the dataset was not the complete cohort. We compared the findings of the analysis with the 3742 cases, to an analysis of the first 1664 women enrolled, and to an analysis conducted only with the subsequent 2078 cases. We found no significant differences in the conclusions drawn from these analyses.

## Results

### Qualitative Accounts of Attempted Murder

#### Strangulation

One of the most prominent themes in accounts of attempted murder was strangulation, as shown by the following examples:

He told me straight that he will kill me. He came back from drinking, he strangled me where I almost died, he stopped when I shouted to stop because there will be no one to care for my kids if I die.

So it happened that I had a huge fight with my partner early this year, this fight was due to infidelity from my partner’s side. I was pregnant and he fought me to a point where he strangled me and beat me up til I started bleeding. I bled until I lost the baby, without him showing signs of helping. This is where I realized that he would have left me to die had the neighbors not stepped in to assist.

“He locked the door and threw the keys under the bed. And came straight to me while I was sitting on the bed. He started choking me while on top of me. I could not move, that’s when I saw a scissor on the table next to the bed, I reached for it and stabbed him with it. That’s how I managed to free myself. I ran outside to ask for help from my neighbor who then took me to the hospital.”

In each of these examples, the participants thought that they could have died but managed to turn the situation around through direct action (e.g. the scissors), appealing to his reason or the intervention of neighbors.

#### Suffocation

Another form of asphyxiation was in the form of suffocation. Several participants described an attempt on their life through suffocation. An interviewer wrote how he had “*suffocated her with a cushion. She couldn’t breathe well. This happened when she was sleeping*.*”* Doing it whilst women were asleep was a common theme among these accounts.

#### Burning

One woman explained *“He almost burned my shack [informal housing, commonly found in crowded townships], but the community managed to stop him*.*”* Another participant described an attempt to kill her from burns, she explained that her partner *“bought cooking oil and then tied me to a chair, boiled the oil and poured it into my whole body*.*”*

Another participant described a lucky escape, that while they were dating, she left to stay with a friend one day, and he came to her friend’s place and burned the shack. Luckily the participant and friend were not there that night, and the shack burned to ashes. Sometimes the risk was clearly to her children as well as to her, as one that she was not home when he tried to burn her house, but her children were inside at the time, *“She [had] tried to talk to him about his violent behavior because it was affecting her children, then he got angry and later that night he set the house alight, not knowing that [she] had gone to the tavern. The neighbors managed to save the kids and firefighters stopped the fire*.*”*

#### Guns

There were few descriptions of the use of guns, largely because the fatality rate of an incident with a gun is much higher than that of an incident where another weapon or strategy is used (Zeineddin et al., 2021). Here were two accounts of the use of guns:

“There was an occasion when they hosted a braai with friends at their place and the participant was busy serving the guests. One of the guests was her boy-friend’s friend, whom at some point, …had an interest in her. Her boyfriend got very angry when she served the friend, claiming that she is giving him some special treatment. The boyfriend then chased everyone out and was left with the participant and beat her up badly and strangled her until she fainted. When she woke up … beat her up again and took out his gun and pulled the trigger. [She] managed to dodge the bullet then it hit the wall”.

Another spoke of how her partner *“One day he tried to shoot me, but I stumbled and fell down, so the bullet missed me and hit the door”*.

#### Stabbing

There were various accounts from participants of being stabbed on occasions when they felt their partner had tried to kill them. One participant related how her boyfriend had come to their house and stabbed her in the chest and then tried to stop her from going to the hospital. Another explained how she thought he would kill her *“…when he stabbed me three times and hit me with an axe on my hand*.*”*

Some women spoke of stabbing as being something that had happened several times. One related that her partner *“stabs her with a knife”* and *“cuts her with a bush knife [machete] at her back”*. Others spoke of being stabbed with broken bottles when they had been drinking, or being threatened with death, as one explained, *“Her boyfriend put a knife under the pillow and threatened to kill her while they were in bed*.*”*

Several participants mentioned the attempted femicide was accompanied by rape. As the following account shows:

He took to the mountain and as we walked, he stabbed me with the knife. He threw a log on my leg… He started beating me and tied my hands with hook wire and started to rape me from front and back. I felt like I was going to die… I was naked and I couldn’t see anything, but I tried and just walked home slowly, also only by grace. When I got home, I fell outside the house and luckily my eldest sister came and found me …After a week I woke up in the hospital.

#### Attempts To Hang the Women

Several participants described incidents with their partners when they attempted to hang them. One explained that her boyfriend had come looking for her at her house, her mother had hidden her under the bed, but he found her and took her with him. When they arrived at his place there was a rope hanging from the ceiling and he instructed her to hang herself with it.

Two participants related similar accounts of their partner putting a rope around their necks and trying to hang them, then escaping when someone intervened. One explained *“He put the rope around my neck to hang me, then my mother came towards us [and she was saved]”*.

#### Blunt Force

There were several accounts of blunt force being used. As one explained *“He beat me up, he hit me with a bottle on my head, and continued to beat me. I was unconscious and so was taken to the hospital”*.

Others just used their fists, as one explained, “*We were drinking at his house. He saw me talking to his friend. He asked me to go to the bedroom with him. That is when he beat me up so bad and he said he wants to kill me”*.

Another participant described how she got a traumatic head injury from her partner’s violence and thought he could have killed her. This is the story the interviewer recorded: *“she went to a wedding and when she came back, he had an axe and a panga, asking her where she had been. The ladies she was with cooled down the situation and when they left, he beat her up and repeatedly banged her head against the floor and did not stop even though she was bleeding. The participant felt the injury affected her brain function because ever since the incident she does not think straight and tends to forget things*.*”*

Another participant explained, *“he once tried to kill me. He took me to the … mountain to kill me. He told me to hang myself, or jump from the top of the mountain. When I refused, he started to beat me up until I begged him to think about my children. Then he left me there, and I walked to the nearest road and asked for help and someone came and took me home*.*”*

#### Designed To Appear as an Accident

Some of the attempts were clearly designed to appear to be accidents or suicide, and may have been recorded as such, or as suicide, had they been successful. A participant described an incident made to look like a railway accident. She explained, “We lived near the railway. He beat me up, hitting me with a gun until I was unconscious. He then dragged me to the railway. I woke up in hospital. I was told that I was rescued by some boys who were passing by, and they stopped him and removed me from the railway.”

Several participants described how their partners tried to kill them through a gas cylinder explosion. As one related, *“There was a time when I wanted to break up with him, he got angry and switched on the gas cylinder to try and hurt me. I had to calm him down and return my bags into our room so that he wouldn’t hurt me*.*”*

Another was nearly drowned, as she related, *“he wanted to push me inside a dam and I had to fight for my life because he was going to just throw me inside that dam”*.

Another’s boyfriend almost drove the car over her and the children. She asserted that she was convinced he was trying to kill her. Another explained how ‘one evening he tried to throw me under a truck in a busy main road.

One participant spoke of an attempt to poison her, she explained, *“He tried poisoning me with something which I’m not sure if it was muthi or poison*.*”* Another related how “he put a drug in my drink, and I fainted and when people interrogated him, he admitted that he put something to kill me.”

### Determining How To Define Attempted Femicide Through a Quantitative Analysis

The analysis was based on the first 3742 women recruited into the study. Table 1 shows their mean age was 30.7 years (range 18–45), 81.0% were Black African and 18.1% were Colored. 7.5% were married, 32.8% were co-habiting, 41.0% had a current boyfriend, and 18.7% women had former relationships. About 76.2% of the women were unemployed. The women were recruited at four sites, in the Western Cape (30.6%), KwaZulu-Natal (29.0%), Gauteng (24.7%) and Limpopo (15.7%). Among these women, 30.2% (1131/3742) reported that there had been a time when their intimate partner had tried to kill them. A further 80 women disclosed that there had been a time when they were in a life-threatening situation, that he had engineered (such as brakes being cut on her car). In total 32.4% (1211/3742) of women recognized and reported to us that there had been an attempt on their life.

Table 2 shows the acts and injuries women had experienced by their own assessment of whether they had experienced a femicide attempt or not. About 21.2% of women had lost consciousness due to a head injury, 19.4% lost consciousness following strangulation, whilst overall 38.5% had been strangled. Burns were reported by 6.3%, and 0.3% of women had been shot with a gun, 10.3% had been smothered, 29.6% had experienced stabs or cuts to the neck, genitals or torso. A further 21.0% had broken bones or teeth, 83.5% bruising, and 15.4% had been admitted to hospital overnight due to their injuries. The bi-variate analysis shows that all these acts and injuries were statistically significantly more common among women who recognized that they had experienced attempted femicide, than among those who did not.

The table also shows the proportion of women who had experienced different acts and injuries who perceived themselves as having experienced attempted femicide. Of the women who experienced loss of consciousness from a head injury, 59.2% perceived they had experienced attempted femicide, as did 63.1% of those who lost consciousness due to strangulation, and 50.5% of those who had been strangled. 91.7% of those who had been shot with a gun perceived themselves to have experienced an attempted femicide, and 55.2% of those who had been smothered and 56.2% of those subjected to burns. 51.8% of those with stabs or cuts to their neck, genitals or torso perceived they had experienced attempted femicide, and 46.3% of those with broken bones and teeth after injuries, and 35.9% of those experiencing bruising. 57.2% of those admitted for one or more nights with injuries also perceived that they had experienced attempted femicide.

**Fig. 1:**
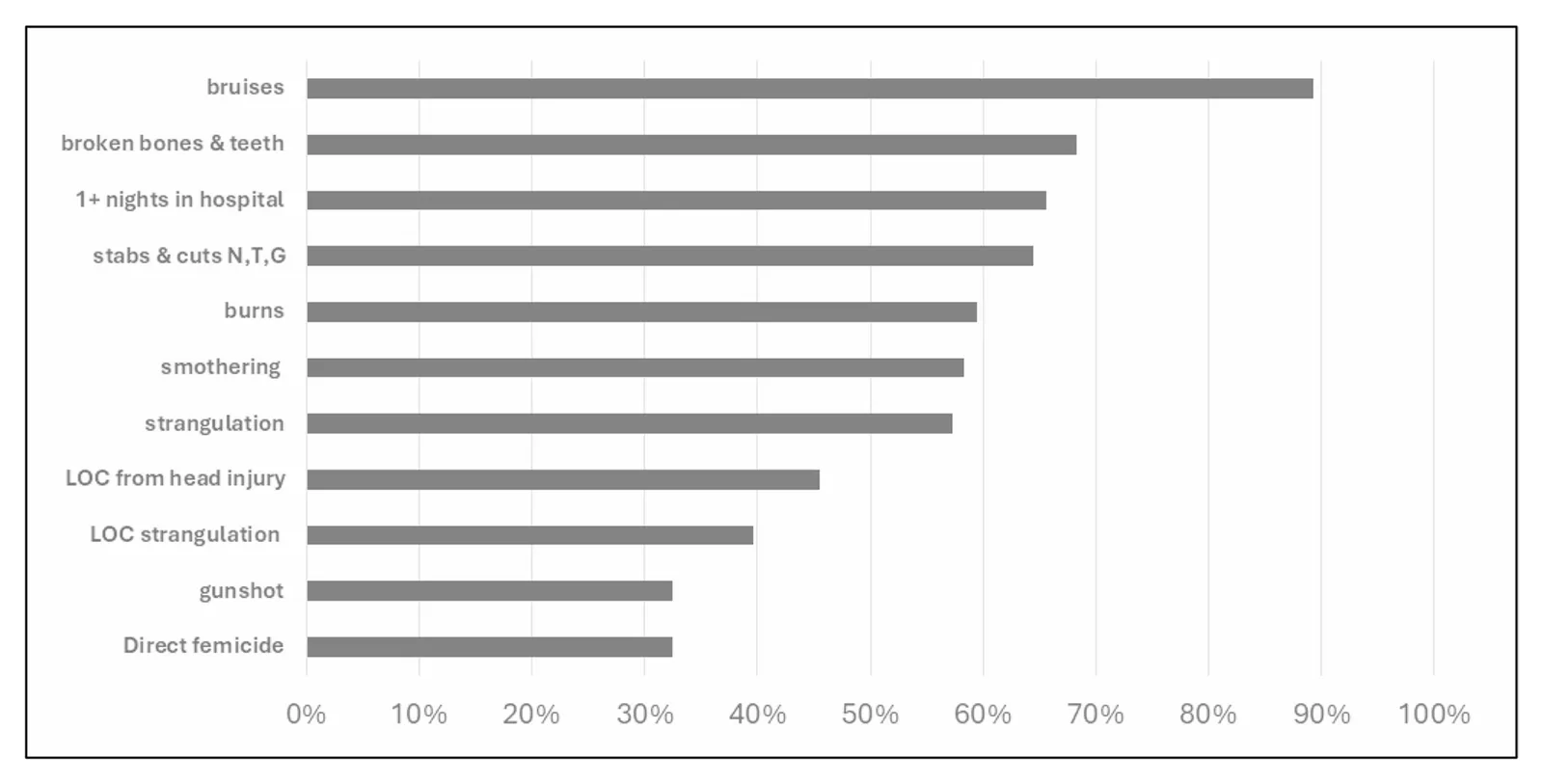
Cumulative percentage of participants potentially classified as having experienced attempted femicide, by different inclusion criteria. Foot-note: LOC = loss of consciousness; N,T, G = neck, torso and genitals

In Fig. 1, we present the proportion of participants who would be considered to have experienced attempted femicide, where different acts or injuries were included in the definition. A third of participants had experienced a direct attempted femicide, and if gunshot injuries were included, the proportion experiencing attempted femicide would be unchanged. Including loss of consciousness from strangulation and head injury would increase it to 39.6% and 45.5%, respectively. Including all strangulation, would increase it to 57.3% and smothering, increases it to 58.3%. Including burns and stabs and cuts to the neck, genitals and torso adds cases to increase it to 59.4% and 64.4% of the sample, respectively. If spending a night or more in hospital due to injuries was included, there is a small increase in the proportion of participants with attempted femicide (taking it to 65.6%). Similarly adding broken bones and teeth only makes a small difference (to 68.3%). Including bruises in the definition, would take the proportion experiencing attempted femicide to 89.3% of the sample.

Table 3 presents a multivariable logistic regression model of potential predictors of a directly recognized attempted femicide. It shows that the strongest associations were for gunshot injuries and loss of consciousness due to strangulation with adjusted odds (aOR) ratios of 9.81 (95% CI 1.22–79.22) and 2.72 (95% CI 2.24–3.31), respectively. All of the injuries from burns, strangulation, bruises, stabs to the neck, genitals and chest, and loss of consciousness from a head injury were associated with a woman perceiving themselves as having experienced attempted femicide. Having spent one or more nights in hospital was associated with a 1.5x increased likelihood of participants perceiving that they had had a femicide attempt. The association was weaker for smothering, aOR 1.27 (95% CI 0.99–1.63) and p-value 0.056, which was narrowly non-statistically significant at the 5% level. Experience of broken bones and teeth was not significantly associated with having experienced a directly perceived femicide attempt, aOR 1.12 (95% CI 0.93–1.36) *p* = 0.233. For that reason, we did not consider broken bones and teeth in further analysis.

To assess the sensitivity and specificity of different approaches to defining attempted femicide, we conducted an analysis where we putatively considered recognized femicide attempts to be a gold standard, and calculated the sensitivity and specificity of different elements to the attempted femicide definition in predicting these cases (Table 4). The first model only included burns and had a high specificity (95.9%) but very low sensitivity (10.9%). In the second model, strangulation was added to burns, and this resulted in the sensitivity rising, and specificity falling, to 63.5% and 69.8% respectively. In the third model, gunshot was added, with very little change in sensitivity and specificity (as expected as there were few). In the fourth model smothering was added, and this somewhat increased the sensitivity (to 66.1%) but reduced the specificity (to 67.7%). In the fifth model, stabs were included, and this increased the sensitivity to 77.7%, but reduced the specificity to 58.2%. Then in model 6, loss of consciousness due to strangulation was added, and this raised the sensitivity to 81.8% and reduced specificity to 55.9%. In model 7, loss of consciousness due to head injury was added, and this raised the sensitivity to 85.0% and further dropped the specificity to 52.7%. In the last model, model 8, having been admitted to hospital for one or more nights due to injury was added, and this raised the sensitivity to 86.1% and dropped the specificity to 50.9%. In model 9, bruises were added. This raised the sensitivity to 97.1% but dropped the specificity to 16.9%.

The ROC AUC, the balance between sensitivity and specificity, and overall performance indicator of the binary classification model. The higher the ROC AUC, the better the performance. Findings suggest that the best balance between sensitivity and specificity is achieved both options 7 and 8 with a ROC AUC of 0.671, which is the highest value among the different models.

## Discussion

Attempted femicide, whether recognized by women or measured through indirect means, had been experienced by between one-and two-thirds of our sample, who were all women who experienced severe IPV. The qualitative accounts of femicide attempts show a diverse range of ways in which male partners had attempted to kill women enrolled in the study. Some of these are clearly accounts of surviving severe injuries, while others are tales of narrow escapes, where the woman may not have been injured at all. Some of them clearly threatened her children as well. In all these accounts, women perceived their lives to have been threatened. However, as Nicolaidis et al. found (Nicolaidis et al., 2003), our research has shown that many women experience injuries that could have been life-threatening, without recognizing them as having constituted an attempt on their life. For multiple reasons women do not recognize their risk, in particular because of the cognitive distortions characteristic of traumatic bonding (Painter SL & Dutton, 1985), may cause women to minimize their risk, deny their partner’s intentions and defend him from criticism. Thus, women’s perception of attempted femicide is not entirely reliable, as it underestimates their real degree of risk. Using the language of sensitivity and specificity, it is very sensitive, in that we do not consider that women in our study will have falsely imagined that their partner tried to kill them, but it is not very specific, in that many women will have faced attempted murder without recognizing themselves as having been at this degree of risk. Our aim in seeking a broader definition was thus to find a definition that would be more specific without losing much sensitivity.

From this starting point, we assessed the associations between a range of indicators of acts that are known to be part of femicide attempts. We selected the acts both because women described them as such, and because they reflect injuries of women murdered by an intimate partner i.e. strangulation, smothering, gunshot wounds and burns (Abrahams et al., 2009). Both stabbing and blunt trauma are prominent mechanisms of death in intimate femicides, however they present a problem in attempted femicide definitions because they occur on a spectrum from small cuts/blunt injuries that would be very infrequently fatal, to stabbing of the neck or chest, or bludgeoning the head, that would be frequently so (Mathews et al., 2009). We have therefore restricted the measure of stabbing to neck, torso and genitals, and investigated whether we might use hospitalization as an indicator of injury severity. We have learned from working with women in this study that many do not access health care for their injuries because they are either prevented from doing so, or they fear being asked about their injuries and their partner being arrested. However, women with very severe injuries more often access health care and are admitted overnight, so we considered whether admission to hospital might be a useful indicator of severity.

We have shown that the proportion of the sample that would be considered to have had a femicide attempt increased incrementally with additional elements to the definition, with the greatest additions coming from loss of consciousness from a head injury and strangulation. Together these nearly doubled the proportion of the sample deemed to have experienced attempted femicide. Other variables only increased the proportion incrementally, until the addition of bruising, which had a greater impact, being experienced by nine out of ten women in the sample. Acknowledging that many acts and injuries co-occur, we built a multi-variable logistic regression model of factors associated with direct femicide attempts and showed that all the acts and injuries - except broken bones and teeth - were associated with these. We found marginal significance for smothering, but have retained this, as it is well-recognized as a mechanism of death in murder and was mentioned in the qualitative accounts.

In the final stage of the analysis, we investigated the impact of different elements of the definition on sensitivity and specificity, when compared to direct femicide attempts, as a putative gold standard (because the sensitivity is so high). Because acknowledged attempted femicide under-counts cases, but we have this valued sensitivity over specificity. The analysis concluded that the ROC was optimized in models 7 and 8, and in the interests of parsimony we suggest that the best definition for our data is model 7. Our findings suggest that a working definition of attempted femicide (as per model 7) recognized attempts on the women’s lives as perceived by women, injuries of a severity leading to loss of consciousness, and acts of strangulation, suffocation, gunshots, burns, and stabbing aimed at the neck, torso or genitals. This has large overlaps with the definition of Campbell et al. (J.C. Campbell et al. 2003a, 2003b), but the advantage of all of the elements being clearly defined, and ill-defined terms such as ‘trauma’ and ‘other severe injuries’ are avoided. The predictive power of this definition in terms of repeated femicide attempts will be tested prospectively in the research.

Hospitalization provided some additional data as a proxy for the severity of injuries, but on its own, it was not a good proxy for attempted femicide as some of these happened in ways where the women escaping did not incur severe injuries. In South Africa generally, public sector hospital beds are only allocated when there is considerable need, but at times it is known that busy trauma departments will admit patients requiring non-life-saving surgery, e.g. to fix a broken arm, so that they can do it at a time when they are not too busy. Furthermore, some acts that can prove fatal, such as smothering or strangulation, often when non-fatal, do not leave any (severe) physical injuries.

### Limitations

Our study represents the first time, to our knowledge, that an attempt has been made to develop an operational definition of attempted femicide, from data available on acts, injuries and healthcare use, as well as direct reports, that could be used in epidemiological research without verification of reports. We recognize that attempting to define attempts is challenging because many women do not recognize the risk they face (or become desensitized after surviving repeated attempts), some attempts do not result in injury, and often injured women do not seek medical assistance. Furthermore, without full information from medical records, we have no way of knowing exactly which women were injured in a manner that threatened their lives, we recognize that some acts may have been applied in a way that would not have been life-threatening, for example burns could have been with a cigarette to a limb, but we do not have further information to distinguish these. We have undertaken a limited sensitivity analysis and did not see that it improved the definition. We only have information on self-reported acts and injuries and there may be some under-reporting. We also recognize that not all potentially fatal acts cause any injury and we are not able to adjudicate the severity of non-(physically) injurious acts. Also, we have omitted a question on attempted hanging. Thus, we recognize that our definition is imperfect.

## Conclusions

This paper presents harrowing accounts of attempted murder and describes one of the first efforts to define attempted femicide for use in research. We acknowledge the challenges in so doing, not least because so many women do not recognize the risk they face from their partners, or else deny it as part of the process of rationalizing to themselves why they stay in very violent relationships. We have shown that it is possible to approach generating a definition of attempted femicide scientifically. The result is quite complex as would be anticipated by the subject in question, but our proposal for a definition is supported by our data. This is an important step forward and lays a foundation for further research which can measure whether women have experienced attempted femicide. Having such a definition can open lines of inquiry about circumstances of risk of femicide which is critical for generating an in-depth, multi-country understanding of the problem, and foundational for efforts to escalate femicide prevention globally.

## Figures and Tables

**Table 1:** Description of the sample

	N=3,742
Mean age (SD)	30.7 (6.7)
Woman’s age group	
<=25 years	969 (25.9%)
26-30 years	876 (23.4%)
31-35 years	902 (24.1%)
36-40 years	710 (19.0%)
41-45 years	285 (7.6%)
Race	
Black African	3,032 (81.0%)
Colored	677 (18.1%)
Indian	8 (0.2%)
White	10 (0.3%)
Mixed race/other	15 (0.4%)
Education	
Incomplete or no high schooling	2,580 (69.0%)
High school completed (passed)	1,161 (31.0%)
Relationship with perpetrator	
Husband	279 (7.5%)
Former husband	35 (0.9%)
Co-habiting partner	1,229 (32.8%)
Former co-habiting partner	242 (6.5%)
Boyfriend	1,536 (41.0%)
Former boyfriend	421 (11.3%)
Occupation	
Unemployed	2,851 (76.2%)
Student/unpaid volunteer	228 (6.1%)
Business	166 (4.4%)
Employed	496 (13.3%)
Province	
Western Cape	1,144 (30.6%)
Limpopo	588 (15.7%)
Kwazulu-Natal	1,085 (29.0%)
Gauteng	925 (24.7%)

**Table 2:** Act and injuries sustained from intimate partner violence, and the association between being subjected to the acts or injuries and recognizing the experience as attempted femicide

Variable	Response	*N*=3742 *n* (col %)	Recognized femicide attempt: *n*=1211 (32.4%) *n* (row %)	None recognized: *n*=2531 (67.6%) *n* (row%)	Odds ratio (95% CI)	*P* value
Lost consciousness due to a head injury	No	2,950 (78.8%)	742 (25.2%)	2,208 (74.9%)	Ref	
	Yes	792 (21.2%)	469 (59.2%)	323 (40.8%)	4.32 (3.67-5.09)	<0.001
Lost consciousness due to being strangled	No	3,015 (80.6%)	752 (24.9%)	2,263 (75.1%)	Ref	
	Yes	726 (19.4%)	458(63.1%)	268 (36.9%)	5.14 (4.33-6.11)	<0.001
Strangulation	No	2,302 (61.5%)	484 (21.0%)	1,818 (79.0%)	Ref	
	Yes	1,440 (38.5%)	727 (50.5%)	713 (49.5%)	3.83 (3.32-4.42)	<0.001
Gun shot	No	3,730 (99.7%)	1,200 (32.2%)	2,530 (67.8%)	Ref	
	Yes	12 (0.3%)	11 (91.7%)	1 (8.3%)	29.19 (2.99-179.84)	0.003
Smothering	No	3,358 (89.7%)	999 (29.8%)	2,359 (70.3%)	Ref	
	Yes	384 (10.3%)	212 (55.2%)	172 (44.8%)	2.91 (2.35-3.61)	<0.001
Burns	No	3,507 (93.7%)	1,079 (30.8%)	2,428 (69.2%)	Ref	
	Yes	235 (6.3%)	132 (56.2%)	103 (43.8%)	2.88 (2.21-3.77)	<0.001
Broken bones or teeth	No	2,956 (79.0%)	847 (28.7%)	2,109 (71.4%)	Ref	
	Yes	786 (21.0%)	364 (46.3%)	422 (53.7%)	2.15 (1.83-2.52)	<0.001
Bruising	No	616 (16.5%)	89 (14.5%)	527 (85.6%)	Ref	
	Yes	3,126 (83.5%)	1,122 (35.9%)	2,004 (64.1%)	3.32 (2.62-4.20)	<0.001
Stabs/cuts to the neck, genitals or torso	No	2,634 (70.4%)	637 (24.2%)	1,997 (75.8%)	Ref	
	Yes	1,108 (29.6%)	574 (51.8%)	534 (48.2%)	2.92 (2.53-3.37)	<0.001
Any overnight hospital admission due to injury in the past year	No	3,165 (84.6%)	881 (27.8%)	2,284 (72.2%)	Ref	
	Yes	577 (15.4%)	330 (57.2%)	247 (42.8%)	3.46 (2.89-4.16)	<0.001

**Table 3:** Multivariable logistic regression model of being subjected to the indicator acts and injuries and recognizing themselves as having experienced a femicide attempt

	Odds ratio (95% CI)	*P*>|z|
Burns	1.77 (1.31–2.40)	< 0.001
Strangulation	1.87 (1.58–2.22)	< 0.001
Gunshot	9.81 (1.22–79.22)	0.032
Smothering	1.27 (0.99–1.63)	0.056
Stabs to neck, genitals & torso	1.84 (1.55–2.18)	< 0.001
Loss of consciousness due to strangulation	2.72 (2.24–3.31)	< 0.001
Loss of consciousness due to head injury	1.94 (1.60–2.35)	< 0.001
Hospital admission 1 night +	1.64 (1.33–2.03)	< 0.001
Broken bones & teeth	1.12 (0.93–1.36)	0.233
Bruises	1.73 (1.34–2.25)	< 0.001

**Table 4:** Analysis of the sensitivity and specificity of different elements of an attempted femicide definition when compared to a putative gold standard of a recognized femicide attempt

	Model 1	Model 2	Model 3	Model 4	Model 5	Model 6	Model 7		
	1. burns	1. burns	1. burns	1. burns	1. burns	1. burns	1. burns		
		2. strangulation	2. strangulation	2. strangulation	2. strangulation	2. strangulation	2. strangulation	2. strangulation	2. strangulation
			3. gunshot	3. gunshot	3. gunshot	3. gunshot	3. gunshot	3. gunshot	3. gunshot
				4. smothering	4. smothering	4. smothering	4. smothering	4. smothering	4. smothering
					5. stabs to the neck, genitals & torso	5. stabs to the neck, genitals & torso	5. stabs to the neck, genitals & torso	5. stabs to the neck, genitals & torso	5. stabs to the neck, genitals & torso
						6. loc strangulation*	6. loc strangulation*	6. loc strangulation*	6. loc strangulation*
							7. loc head injury*	7. loc head injury*	7. loc head injury*
								8. hospital admission 1 + night	8. hospital admission 1 + night
									9. bruises
Prevalence of recognized femicide	32.36	32.36	32.36	32.36	32.36	32.36	32.36	32.36	32.36
Sensitivity	10.90	63.50	63.75	66.14	77.7	81.75	84.97	86.13	97.11
Specificity	95.93	69.77	69.74	67.68	58.16	55.87	52.71	50.93	16.87
Positive Predictive Value	56.17	50.13	50.2	49.47	47.05	46.99	46.23	45.65	35.85
Negative Predictive Value	69.23	79.98	80.08	80.69	84.5	86.48	87.99	88.47	92.42
ROC AUC^#^	0.627	0.651	0.651	0.651	0.658	0.667	0.671	0.671	0.641

* *loc* loss of consciousness;

# *ROC AUC* receiver operating characteristics area under curve

## Data Availability

Data are available on request from the authors.
